# Think BIG, Stay Connected: A Regional Immunology Meeting as a Durable Model for Scientific Community Building

**DOI:** 10.1111/imcb.70165

**Published:** 2026-09-29

**Authors:** Danielle I. Stanisic, Rajiv Khanna, Severine Navarro, J. Alejandro Lopez

**Affiliations:** ^1^ Institute for Biomedicine and Glycomics Griffith University Southport Queensland Australia; ^2^ QIMR Berghofer Medical Research Institute Brisbane Queensland Australia; ^3^ School of Environment and Sciences Griffith University Nathan Queensland Australia

**Keywords:** communities of practice, early‐career researchers, immunology, mentoring, regional networks, scientific meetings

## Abstract

Regional scientific meetings occupy a distinctive but underexamined position within the research ecosystem. Smaller and more accessible than national congresses, yet broader in scope than institutional seminar programm, they provide opportunities for repeated interactions across institutions, career stages, and research domains. The Brisbane Immunology Group (BIG) meeting is one of the longest‐running and most influential regional meetings associated with the Australian and New Zealand Society for Immunology (ASI), providing a focal point for immunology researchers across Queensland, Australia, and beyond. This article examines the BIG meeting as a case study of a regional immunology community that has sustained scientific engagement, mentoring, and collaboration for 25 years. Drawing on historical records and thematic analysis of interviews with organizers, senior contributors, and recent attendees, we identify six characteristics that have contributed to the longevity and impact of the meeting: accessibility, cross‐institutional ownership, intergenerational participation, social integration, programmatic continuity with adaptive innovation, and deliberate recognition of community contributions. We argue that BIG functions not merely as an annual scientific meeting but as a regional community of practice that supports scientific collaboration, professional identity formation, and leadership development. The BIG experience provides broader lessons regarding the role of regional meetings in strengthening scientific communities, supporting future scientific leaders and sustaining disciplinary vitality.

## Graphical abstract

Evolution of BIG meeting booklet covers used for meeting invitation, promotion, and organisation over time.

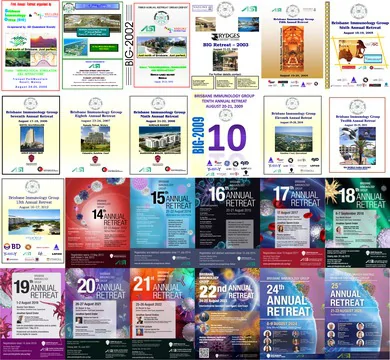

## 1 Introduction

Scientific meetings are a pervasive and enduring feature of modern science. Although traditionally viewed as vehicles for the dissemination of new findings, conferences play a much broader role within research communities. Meetings facilitate knowledge exchange, networking, mentoring, collaboration, professional development, and the formation of scientific identity. Increasingly, studies of scientific conferencing have suggested that some of the most valuable outcomes of conferences arise not from formal presentations but from the social and professional interactions that occur around them, generating collaborations, partnerships, mentoring relationships, and future research opportunities. Conferences therefore function not only as sites of information transfer but also as mechanisms for generating scientific and social capital [1, 2, 3].

Historians and sociologists of science have argued that scientific meetings play a more active role in disciplinary development than is often recognized. Rather than merely reflecting the progression of a field, meetings help create and shape disciplines by bringing together researchers around emerging problems, establishing intellectual leadership, defining research priorities, and facilitating the development of scientific communities. Analyses of international immunology meetings during the second half of the twentieth century demonstrated that meetings contributed significantly to the emergence of modern immunology, the formation of subdisciplines, and the integration of previously peripheral fields into mainstream immunological science. In this view, conferences serve not simply as mirrors of scientific progress but as engines of disciplinary development [4, 5].

Despite extensive scholarship on scientific communication and conferencing, relatively little attention has been devoted to the role of sustained regional meetings in building long‐term scientific communities. Existing studies have focused predominantly on major national and international congresses; virtual and hybrid conferencing models; sustainability, equity, and inclusion; or structured mentoring initiatives. Consequently, there remains limited understanding of how smaller regional meetings contribute to scientific integration, collaboration, mentoring, and community cohesion over extended periods. This gap is particularly relevant in geographically dispersed research environments where repeated face‐to‐face interactions may otherwise be limited, such as in Australia [1, 2].

Recent discussions about the future of scientific conferencing have highlighted characteristics associated with successful meetings. These include affordability and accessibility, opportunities for informal networking, participation across diverse career stages, structured opportunities for early‐career researchers, inclusivity, cross‐disciplinary interaction, and mechanisms that support engagement beyond the formal scientific program. Importantly, a growing body of literature suggests that smaller meetings often provide advantages over larger congresses by lowering barriers to interaction, increasing opportunities for meaningful engagement, and facilitating repeated encounters among participants. Informal interactions, often regarded as secondary to the scientific program, are increasingly recognized as important drivers of collaboration, creativity, and professional development [1, 2, 3].

The Brisbane Immunology Group (BIG) meeting provides an opportunity to explore these questions through the lens of a long‐standing regional scientific community. Established in 2000 as an Australian and New Zealand Society for Immunology (ASI)‐supported initiative, BIG has become one of the most prominent regional immunology meetings in Australia and a key component of the ASI scientific ecosystem. The meeting brings together students, early‐career researchers (ECRs), technical staff, clinicians, industry representatives, and senior investigators from across Queensland, Australia, and beyond. Over 25 years, it has maintained stable participation while adapting its program and organizational structure to meet changing scientific and community needs (Figure 1). Its longevity and sustained success provide a valuable case study of how regional scientific meetings can support collaboration, mentoring, leadership development, professional identity formation, and the long‐term sustainability of scientific communities.

**FIGURE 1:**
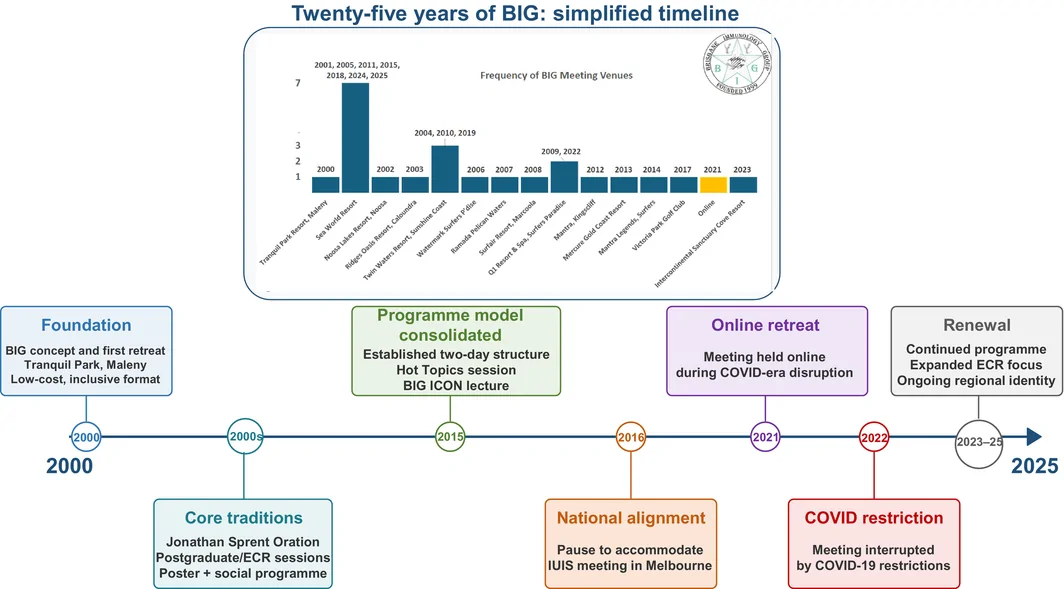
Twenty‐five years of BIG: Establishment, evolution, and adaptation. Simplified timeline summarizing major phases in the development of the Brisbane Immunology Group (BIG) meeting, including its establishment in 2000, evolution of venues, emergence of core traditions, program consolidation, interruptions associated with IUIS and COVID‐19, and subsequent renewal. The figure highlights how BIG has retained a stable community‐building structure while adapting its venues, programme elements and delivery formats in response to changing scientific and organizational circumstances. BIG: Brisbane Immunology Group; ECR: Early Career Researcher; IUIS: International Union of Immunological Societies.

We propose that BIG is most usefully understood not simply as an annual scientific meeting, but as a regional immunology community of practice (Figure 2). Communities of practice emerge when individuals with common interests engage in repeated interactions, develop shared ways of working, and collectively contribute to the development of their field. Through repeated participation, collective stewardship, and intergenerational engagement, BIG has evolved into a community that supports scientific exchange, mentoring, collaboration, leadership development, and professional identity formation [6].

**FIGURE 2:**
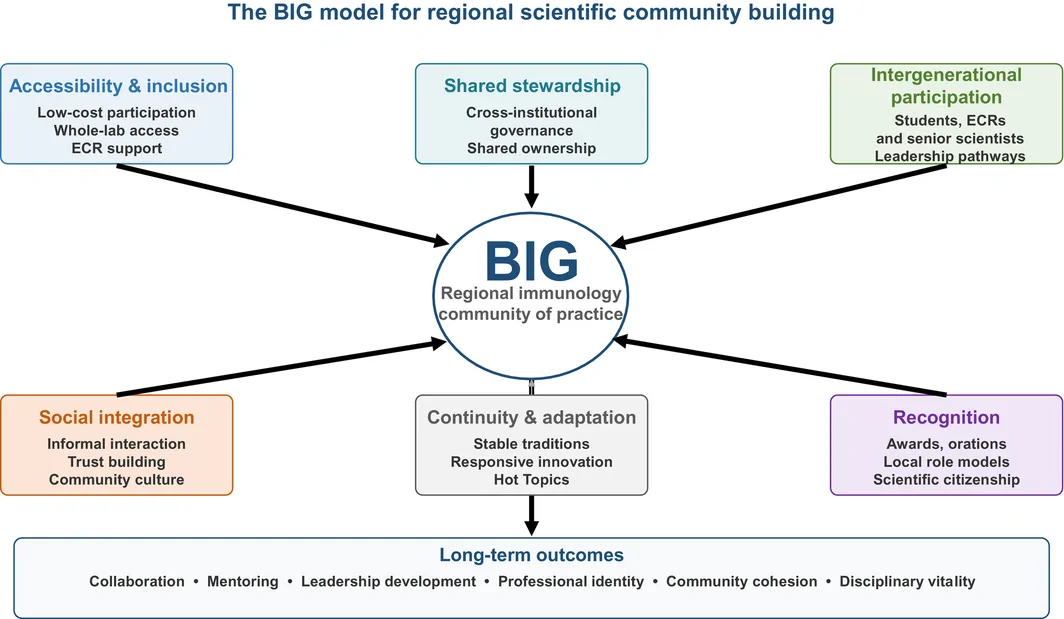
BIG model for regional scientific community building. Conceptual framework summarizing the six interacting mechanisms identified as contributing to the longevity and impact of the Brisbane Immunology Group (BIG) meeting: Accessibility and inclusion, shared stewardship, intergenerational participation, social integration, continuity with adaptation, and recognition. Together, these mechanisms support BIG as a durable regional immunology community of practice, generating long‐term outcomes including collaboration, mentoring, leadership development, professional identity formation, community cohesion and disciplinary vitality. BIG: Brisbane Immunology Group; ECR: Early‐Career Researcher.

Drawing on historical records and thematic analysis of interviews with organizers, long‐standing contributors, and recent attendees, this article addresses four questions. First, what organizational principles have contributed to the sustainability of the BIG meeting over 25 years? Second, how has BIG facilitated mentoring, networking and scientific integration within the Queensland immunology community? Third, what advantages and limitations emerge from a regional meeting model compared with institutional, national and international meetings? Finally, which elements of the BIG model may be transferable to other scientific communities seeking to strengthen participation, collaboration and community cohesion?

By addressing these questions, we seek to move beyond a local historical account and contribute to broader discussions regarding the role of regional scientific meetings in supporting scientific excellence, community formation, and disciplinary development.

## 2 Approach and Sources

This article uses a qualitative case study approach to examine the development and enduring role of the BIG meeting over its 25‐year history. This study was approved by the Griffith University Human Research Ethics Committee. The analysis draws on historical meeting records, program materials, archival documents and interviews with organizers, long‐term contributors, and recent attendees representing a range of career stages and institutions. Selected quotations are included as illustrative evidence of participant perspectives and experiences. Rather than serving as interview transcripts, they are used to exemplify themes that emerged consistently from contributors. The aim of this study was not to provide a comprehensive chronological history of BIG, but to examine how participants understand its contribution to scientific exchange, professional development, and community building within Queensland immunology, and to identify lessons that may be relevant to other regional scientific communities.

## 3 The BIG Model: Themes Emerging From Twenty‐Five Years

### 3.1 Origins and Rationale: Filling the Gap Between Local Seminars and National Conferences

BIG emerged in response to a practical and scientific gap. Institutional seminars provided local opportunities for exchange, whereas the national ASI meeting created broader disciplinary connections but rotated across Australia and therefore returned to Queensland only intermittently. BIG was designed to occupy the intermediate space between these formats: more inclusive and diverse than an institutional seminar, but more accessible, intimate, and locally anchored than a national conference.

My recollection is that meetings involving Brisbane immunologists around the year 2000 were limited to the seminar series run at places like QIMR, IMB at The University of Queensland and the Princess Alexandra Hospital campus. Immunologists found it difficult to travel consistently to these seminars and so a few of us discussed having an annual meeting away from the usual seminar series which would bring immunologists together to socialise and discuss science.Graham Leggatt

At the time, ASI meetings were the only opportunity for immunologists to gather and connect. However, their national rotation meant they only returned to Queensland roughly every ten years, limiting access for many local researchers.Alejandro Lopez

The first BIG retreat was organized in 2000 at Tranquil Park at Maleny, north of Brisbane and lasted one and a half days. Costs were kept down to encourage ECRs to attend.Rajiv Khanna

#### 3.1.1 Accessibility as a Deliberate Scientific Strategy

Affordability has been central to BIG from its inception. It has allowed participation by students, early‐career researchers (ECRs), and complete research groups, rather than restricting attendance to senior investigators or individuals with independent travel budgets. In this sense, accessibility is not simply an administrative feature of the meeting; it is a scientific strategy that broadens participation and increases opportunities for interaction across career stages.

A short format of two days was designed to keep costs low which would hopefully encourage entire lab groups to attend.Graham Leggatt

It's a local and affordable annual meeting that brings together multi‐disciplinary groups from across QLD. It provides great exposure for students and postdocs, while also giving group leaders a chance to explore potential collaborations and funding opportunities.Marcela Montes de Oca

BIG stands out for its grassroots organization, affordability, and strong sense of community. Over the years, the organizing committees have worked hard to keep the BIG meeting costs lower than those of comparable events.Alejandro Lopez

#### 3.1.2 Cross‐Institutional Ownership and Shared Stewardship

A further defining feature of BIG is its cross‐institutional stewardship. The meeting is not owned by a single laboratory, institute, or university. Its organizing committee has drawn from major immunology research organizations in Queensland, ensuring that the program reflects the breadth of the local community and that responsibility for the meeting is distributed across institutions. This shared stewardship helps prevent the meeting from becoming a single‐institution showcase and strengthens the sense of collective ownership.

Because the BIG organizing committee has representatives across all the major QLD research organizations, we have a good understanding of what research is being undertaken and will be suitable for invited presentations.Matt Sweet

The Queensland immunology community has always been interactive and collaborative, but I think BIG meetings can take some credit for facilitating this sense of community.Graham Leggatt

Over the last 2 decades the Queensland immunology community has become more interactive and collaborative. BIG has played a role in that. It has enabled opportunities for shared students and grant funding.Rajiv Khanna

#### 3.1.3 Intergenerational Participation and Leadership Development

BIG has deliberately placed students and ECRs within the center of the program rather than at its margins. Over time, this has created a visible progression from attendee to presenter, award recipient, organizer, group leader, and mentor. Such intergenerational continuity is one of the mechanisms through which the meeting reproduces and renews the community.

BIG is a vibrant and inclusive community of immunologists, which more recently has focused on providing opportunities for students, and ECRs, but with the involvement of senior leaders from across Queensland and interstate invitees.Gabrielle Belz

Initially there was only one session focusing on students/postdocs but now there are 4 sessions. All prizes are now for ECRs, and students.Rajiv Khanna

BIG has a lot of invited presentations from emerging researchers, selected abstracts and awards for postgraduate students/ECRs. So, it gives opportunities to help build careers through awards and invited presentations.Matt Sweet

#### 3.1.4 Scientific Program, Traditions, and Adaptive Innovation

The program has combined continuity with adaptation. Stable elements, such as postgraduate sessions, the Jonathan Sprent Oration, poster sessions, invited speakers, and social events, provide identity and familiarity. At the same time, innovations such as Hot Topics sessions have allowed the meeting to respond to emerging scientific priorities and community feedback.

The program has always deliberately evolved to meet the needs of the retreat, but it is gratifying to see some consistent features such as the Jonathon Sprent Oration and the BIG ICON presentation.Graham Leggatt

The “Hot Topics” session is also unique; invited speakers taking a deep dive into hot and newly emerging areas of immunology and cell biology.Rajiv Khanna

Personally, I hope the BIG meeting stays the same. Its current format has worked so well for so many years. It is approachable, engaging, and fosters a real sense of community. I think there is value in maintaining that consistency.Marcela Montes de Oca

#### 3.1.5 Social Integration and Community Culture

Participants repeatedly identified the social architecture of BIG as central to its success. The meeting integrates science and sociality rather than treating them as separate components. Communal meals, poster sessions, and beach cricket provide settings in which students, ECRs, and senior investigators interact with fewer barriers. These interactions are not incidental to the meeting's purpose; they are part of how the community is built.

BIG has set the standard of branch meetings for the ASI. Its focus on interactivity and openness has made it a formidable platform for the exchange of ideas and the inspiration of students and emerging researchers.Jose Villadangos

Conversations and collaborations benefit from relaxed environments where participants have an opportunity to leave behind, even if only for a couple of days, the pressures and frustrations of everyday work.Jose Villadangos

It also has beach cricket which gets everyone together in a fun, relaxing way beyond the normal networking you see at other conferences.Matt Sweet

#### 3.1.6 Regional Identity and Broader Influence

BIG has also contributed to the identity of Queensland immunology as a coherent community within the national landscape. Contributors described the meeting as a means of strengthening connections within Queensland while creating pathways to national recognition through ASI, visiting speakers, interstate participation, and emerging leaders.

BIG, Queensland and Immunology are a tripod; you cannot remove any of its legs without losing the balance of the whole.Jose Villadangos

The BIG meeting is crucial for sustaining and strengthening immunology in Queensland. It provides a regular meeting point for sharing data, building networks, and mentoring the next generation of researchers.Gabrielle Belz

The BIG meeting plays a substantial role in supporting immunologists within Queensland. I think having it every year is a valuable opportunity for Queensland immunologists to catch up, reconnect, network and collaborate.Wilson Nguyen

## 4 Discussion

### 4.1 BIG as Scientific Infrastructure

Historical analyses of immunology meetings have shown that conferences can play an active role in shaping scientific disciplines rather than simply documenting them. Silverstein and Söderqvist argued that meetings contribute to the formation of intellectual communities, identify scientific leadership, and facilitate the integration of emerging areas into the mainstream of a discipline. Scientific meetings therefore function not only as forums for communication but also as mechanisms through which disciplines evolve and institutionalize themselves [4, 5].

Viewed through this lens, BIG appears to represent a form of regional scientific infrastructure. Although organized as an annual meeting, its longer term significance lies in the relationships, professional networks, and collaborative structures that have emerged around it. Over 25 years, BIG has helped shape the Queensland immunology community by bringing together researchers from multiple institutions, career stages, and scientific backgrounds within a recurring environment of scientific exchange and collegial interaction.

Across the interview material, participants consistently characterized BIG as more than a venue for scientific presentations. Rather, BIG displayed many of the characteristics of a community of practice, including repeated interaction, shared identity, collective stewardship, and intergenerational participation [6]. It was also viewed as a mechanism for fostering collaborations, supporting mentoring relationships, facilitating leadership development, and reinforcing a shared disciplinary identity. These observations suggest that the enduring contribution of BIG may be less about any individual scientific program and more about its role in sustaining a scientific community over time. This interpretation is consistent with the view that scientific meetings act as important sites of disciplinary cohesion and community formation [4, 5].

### 4.2 Generating Social Capital Through Repeated Interactions

Recent studies of scientific conferences increasingly recognize networking and relationship formation as central outcomes of conference participation. Conferences facilitate professional development, research empowerment, and collaboration through interactions that frequently extend beyond the duration of the meeting itself [1, 2, 3].

A recurring theme throughout the interviews was the role of BIG in facilitating collaboration, networking, and professional development. Participants described opportunities for establishing research partnerships, identifying future collaborators, accessing professional advice, and gaining exposure to new scientific perspectives. Such interactions occurred across institutions and career stages, reinforcing the integrative function of the meeting.

The repeated nature of these interactions may be particularly important. Unlike large international congresses, where encounters may be infrequent and transient, BIG allows researchers to reconnect annually over extended periods. Such repeated interactions appear to strengthen trust, facilitate collaboration, and reinforce a sense of collective identity. In this respect, BIG functions as a mechanism for the accumulation of social capital across the Queensland immunology community.

These observations align with broader evidence suggesting that scientific meetings contribute to the development and maintenance of professional networks that support future scientific activity. The accumulation of these relationships may be one of the most significant yet least formally recognized outcomes of regional meetings [1, 2, 3].

### 4.3 Informal Interactions as Drivers of Scientific Collaboration

One of the strongest themes emerging from both the conference literature and the BIG interviews concerns the importance of informal interactions. Participants in recent analyses of scientific conferencing consistently identified spontaneous conversations, social activities, and informal exchanges as among the most valuable components of scientific meetings and among the most difficult aspects to reproduce in virtual environments [1, 2, 3].

The BIG experience strongly supports these observations. Participants repeatedly highlighted the importance of informal interactions in fostering collegiality, collaboration, and a sense of community. Activities such as communal meals, poster sessions, and social events may initially appear peripheral to the scientific program; however, they can also be understood as deliberate mechanisms for reducing barriers between institutions, disciplines, and career stages and for creating opportunities for conversations that might not otherwise occur.

Rather than functioning as recreational additions, these activities appear to constitute part of the meeting's social architecture. The BIG experience suggests that the effectiveness of scientific meetings depends not only on the quality of formal presentations but also on the opportunities they provide for informal engagement. This finding is consistent with contemporary discussions regarding the future of scientific conferencing, which increasingly recognize informal networking as a major contributor to scientific creativity, trust‐building, and long‐term collaboration [1, 2, 3].

The rapid growth of virtual conferences during and after the COVID‐19 pandemic demonstrated important benefits in terms of accessibility, cost, and participation. However, researchers consistently reported that opportunities for spontaneous interaction, informal networking, and community building remained difficult to reproduce in virtual environments [2, 3, 7].

### 4.4 BIG as a Mentoring Ecosystem

The mentoring literature increasingly emphasizes the importance of embedding professional development within broader communities rather than relying exclusively on traditional one‐to‐one mentoring relationships. Effective mentoring systems create opportunities for repeated interactions, peer support, community integration, and networking across institutions and career stages. Professional society mentoring initiatives and peer‐mentoring networks have been shown to provide important complements to traditional hierarchical mentoring structures [8, 9].

The findings presented here suggest that BIG functions as such a mentoring ecosystem. Students, postdoctoral researchers, and established investigators participate in a shared environment that supports multiple forms of mentoring simultaneously. These include traditional senior–junior relationships, peer mentoring among trainees and ECRs, and informal guidance arising through repeated participation over time.

Participants consistently emphasized the value of the meeting in providing visibility for students and ECRs, facilitating interactions with senior investigators and supporting career development. The evolution of the program itself, including the expansion of ECR‐focused sessions and awards, reflects increasing recognition of these functions.

Importantly, mentoring within BIG extends beyond scientific training. The meeting provides opportunities for networking, leadership development, professional socialization, and integration into the broader immunology community. Consequently, BIG may be viewed not merely as a venue for scientific dissemination but as a mechanism for developing future scientific leaders and strengthening the continuity of the discipline. This broader conception of mentoring is consistent with contemporary models that emphasize communities, networks, and multiple mentoring relationships rather than traditional hierarchical arrangements alone [6, 8, 9].

### 4.5 Advantages of the Regional Meeting Model

The BIG experience highlights several advantages associated with regional scientific meetings. Compared with institutional seminar programs, regional meetings provide greater scientific diversity, broader opportunities for collaboration, and increased exposure to researchers working across different organizations and specializations. Compared with national and international congresses, regional meetings are generally more accessible financially and geographically, and often enable participation by entire research groups rather than individual representatives.

Accessibility emerged as a particularly important theme in the interviews. This observation aligns closely with recent analyses of scientific conferencing, which identify cost, travel, and time commitments as significant barriers to participation, particularly for students and ECRs [1, 2, 3]. Regional meetings may therefore play an important role in broadening participation and reducing structural barriers to professional engagement.

Importantly, participation in BIG appears not to be restricted to established scientific leaders. The deliberate inclusion of technical staff, students, and ECRs distinguishes the meeting from many specialist workshops and elite conferences. Such inclusiveness may contribute significantly to community cohesion, facilitate knowledge exchange across career stages, and strengthen the overall capacity of the local scientific ecosystem.

These observations are also consistent with the concept of communities of practice, in which participation is not restricted to recognized experts but encompasses individuals at different stages of professional development who contribute to and learn from a shared community [6].

### 4.6 Limitations and Challenges

Although the BIG model offers many strengths, several challenges should also be acknowledged. Its success has relied heavily on volunteer effort, institutional goodwill, and sustained organizational leadership. As venue costs and logistical requirements continue to increase, maintaining affordability may become increasingly difficult.

Close‐knit scientific communities inevitably face the risk of insularity. Although strong networks can promote trust and collaboration, they may also unintentionally limit diversity of participation or create barriers for newcomers if deliberate efforts are not made to maintain inclusivity and renewal. Continued attention to gender equity, institutional representation, emerging research areas, and participation by new members will remain important.

Broader changes in scientific conferencing are creating new expectations regarding accessibility, hybrid participation, family responsibilities, equity, and environmental sustainability. The COVID‐19 pandemic demonstrated that virtual and hybrid meetings can reduce costs, increase accessibility and broaden participation, particularly for individuals who may face barriers to attending in‐person meetings [2, 3, 7, 10]. At the same time, studies consistently emphasize that virtual formats are less effective at facilitating informal networking, spontaneous interactions and the development of professional relationships that often underpin collaboration and mentoring [2, 3, 7, 10]. The challenge for regional meetings such as BIG will be to respond to these evolving expectations while preserving the face‐to‐face interactions that participants consistently identify as central to the meeting's value. Finally, the present study is based primarily on qualitative reflections and historical records. Future work could strengthen understanding of regional scientific communities through social network analysis, longitudinal tracking of collaborations, participant surveys and evaluation of career outcomes associated with meeting participation. Such approaches would provide a more systematic evaluation of how regional scientific meetings contribute to scientific development, leadership formation, and disciplinary cohesion.

### 4.7 Transferable Principles for Regional Scientific Communities

The BIG experience suggests a number of principles that may be relevant to other scientific communities seeking to strengthen collaboration, mentoring, and community engagement (Table 1). These include maintaining affordability and accessibility, fostering shared governance across institutions, providing visible support for students and ECRs, integrating scientific and social activities, balancing continuity with adaptive innovation, and recognizing both scientific and community contributions.

**TABLE 1:** Transferable principles for regional scientific communities.

Principle	Application
Accessibility through low‐cost participation	A regional meeting is most effective when students, ECRs, and complete research groups can attend without prohibitive financial barriers.
Shared governance across institutions	Cross‐institutional organizing committees distribute ownership, reduce institutional bias, and help identify emerging research and emerging researchers.
Visible support for students and ECRs	Dedicated talks, poster sessions, awards, and informal access to senior researchers create pathways for confidence, credibility, and leadership.
Integration of scientific and social activities	Social interaction should be treated as part of meeting design because informal exchanges often generate trust, collaboration, and mentoring.
Stability combined with adaptive innovation	Recognizable traditions support identity, whereas new formats such as Hot Topics sessions sustain scientific relevance.
Recognition of scientific and community contributions	Named lectures, awards, and local honors signal what the community values and create visible models of scientific citizenship.

Although developed within the context of Queensland‐based immunology research, these principles are unlikely to be unique to immunology. Rather, they are consistent with broader evidence regarding the characteristics of successful scientific meetings, mentoring environments and communities of practice [1, 2, 6, 8]. Collectively, they suggest that regional meetings can make important contributions to scientific development not despite their smaller scale, but because that scale permits repeated interaction, accessibility and community formation [1, 2, 6].

## 5 Conclusion

Over 25 years, the BIG meeting has evolved from a regional retreat into a durable scientific community of practice. Its success appears to derive not from any single program element but from a combination of accessibility, cross‐institutional ownership, intergenerational participation, social integration, and adaptive continuity.

The broader lesson emerging from BIG is that regional scientific meetings can fulfill roles that extend far beyond scientific communication. They can support mentoring, generate social capital, strengthen disciplinary identity, and facilitate collaborations that shape the scientific landscape over decades. In doing so, they become more than annual meetings; they become enduring scientific infrastructure.

As pressures on traditional conference models continue to increase, the BIG experience provides evidence that smaller, inclusive, and community‐oriented meetings remain highly relevant. Indeed, their capacity to integrate scientific excellence with community building may make them increasingly important for the future development of scientific disciplines.

## Author Contributions

**Danielle I. Stanisic:** conceptualization, writing – original draft, writing – review and editing. **Rajiv Khanna:** conceptualization, writing – review and editing. **Severine Navarro:** conceptualization, writing – original draft, writing – review and editing. **J. Alejandro Lopez:** conceptualization, writing – original draft, writing – review and editing.

## Conflicts of Interest

All authors have served as members of the BIG meeting organizing committee.

## Data Availability

Data sharing not applicable to this article as no datasets were generated or analysed during the current study.
